# Supplementary material for: A mitotic CDK5-PP4 phospho-signaling cascade primes 53BP1 for DNA repair in G1
Source: Nat Commun. 2019 Sep 18;10:4252. doi: 10.1038/s41467-019-12084-x (PMC6751209; doi:10.1038/s41467-019-12084-x)
Supplement: Supplementary file 1 — Supplementary Information [file 41467_2019_12084_MOESM1_ESM.pdf]

**SUPPLEMENTARY INFORMATION**

# A mitotic CDK5-PP4 phospho-signaling cascade primes 53BP1 for DNA repair in G1

Xiao-Feng Zheng, Sanket Acharya, Katherine N Choe, Kumar Nikhil, Guillaume O Adelmant, Shakti R Satapathy, Samanta Sharma, Keith Viccaro, Sandeep Rana, Amarnath Natarajan, Peter Sicinski, Jarrod A Marto, Kavita Shah, Dipanjan Chowdhury

**Contents:**

**Supplementary Figure 1 (Related to Figure 1): PP4R3β S840 is a conserved residue that is phosphorylated in mitosis.**

**Supplementary Figure 2 (Related to Figure 2): Phospho-antibodies specifically recognize 53BP1 T1609/S1618 and PP4R3β S840 in mitotic cells.**

**Supplementary Figure 3 (Related to Figure 2 and Figure 3): CDK5 is active mitosis and required for irradiation-induced 53BP1 foci formation in G1.**

**Supplementary Figure 4 (Related to Figure 3 and 4): Chemical genetic system for specific inhibition of CDK5.**

**Supplementary Figure 5: CDK5 binding partner KIAA0528 is required for the recruitment of 53BP1 to DNA damage in non-neuronal cells.**

**Supplementary Table 1: List of primers used in this study.**

**Supplementary Table 2: List of antibodies used in this study.**

## Supplementary figure 1 a-f

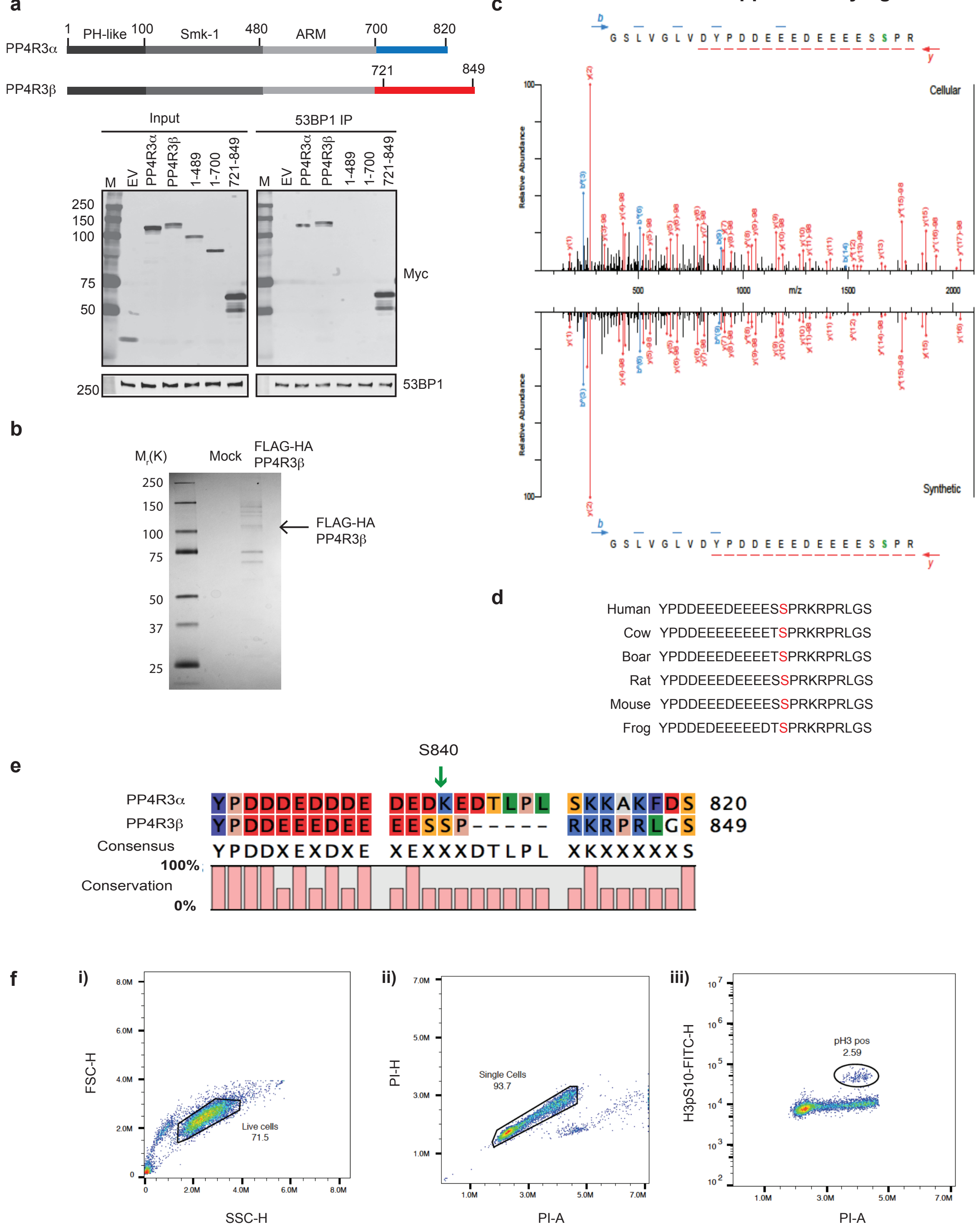

**Supplementary figure 1g**

**g**

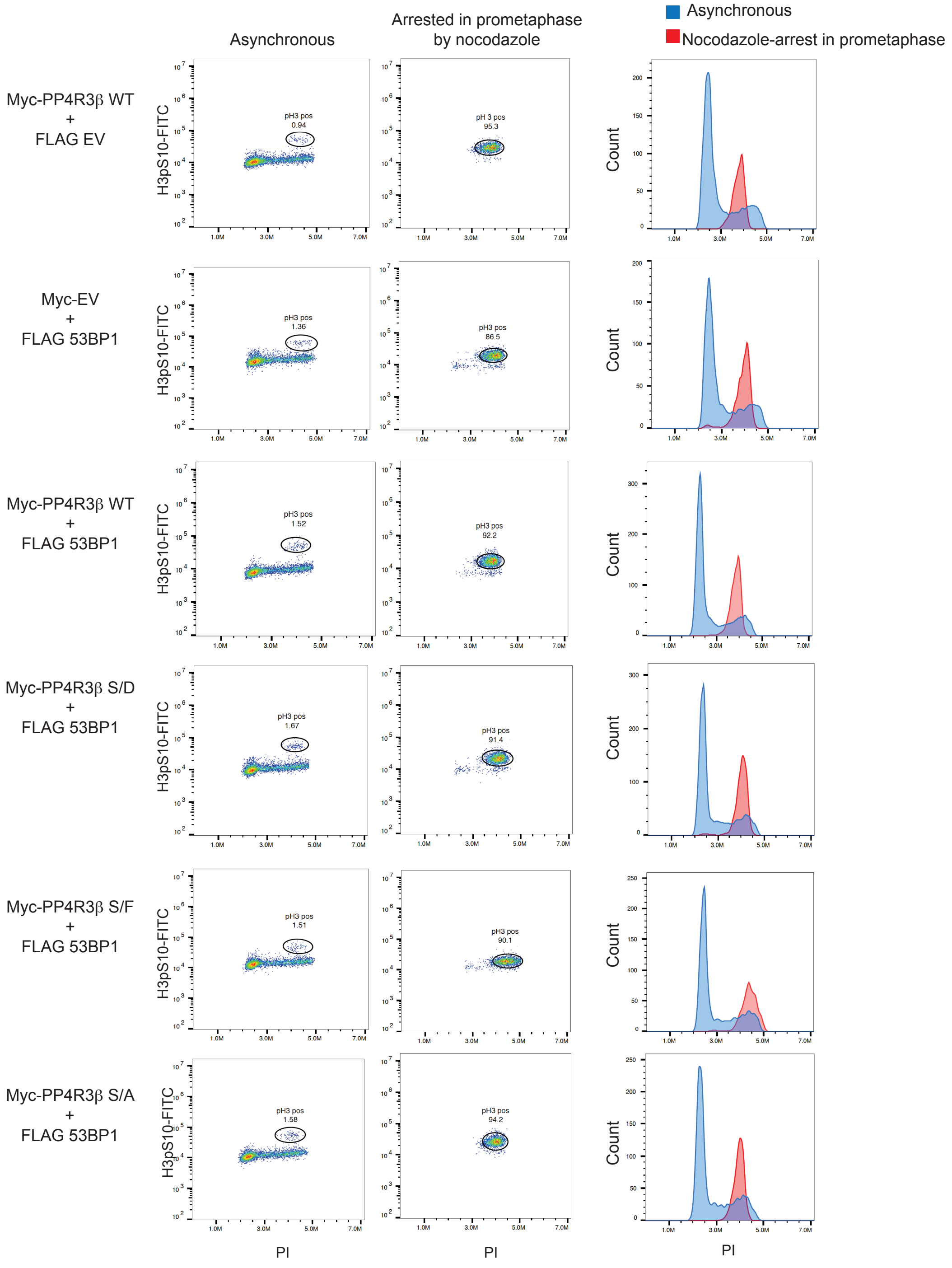

## Supplementary figure 1 h-j

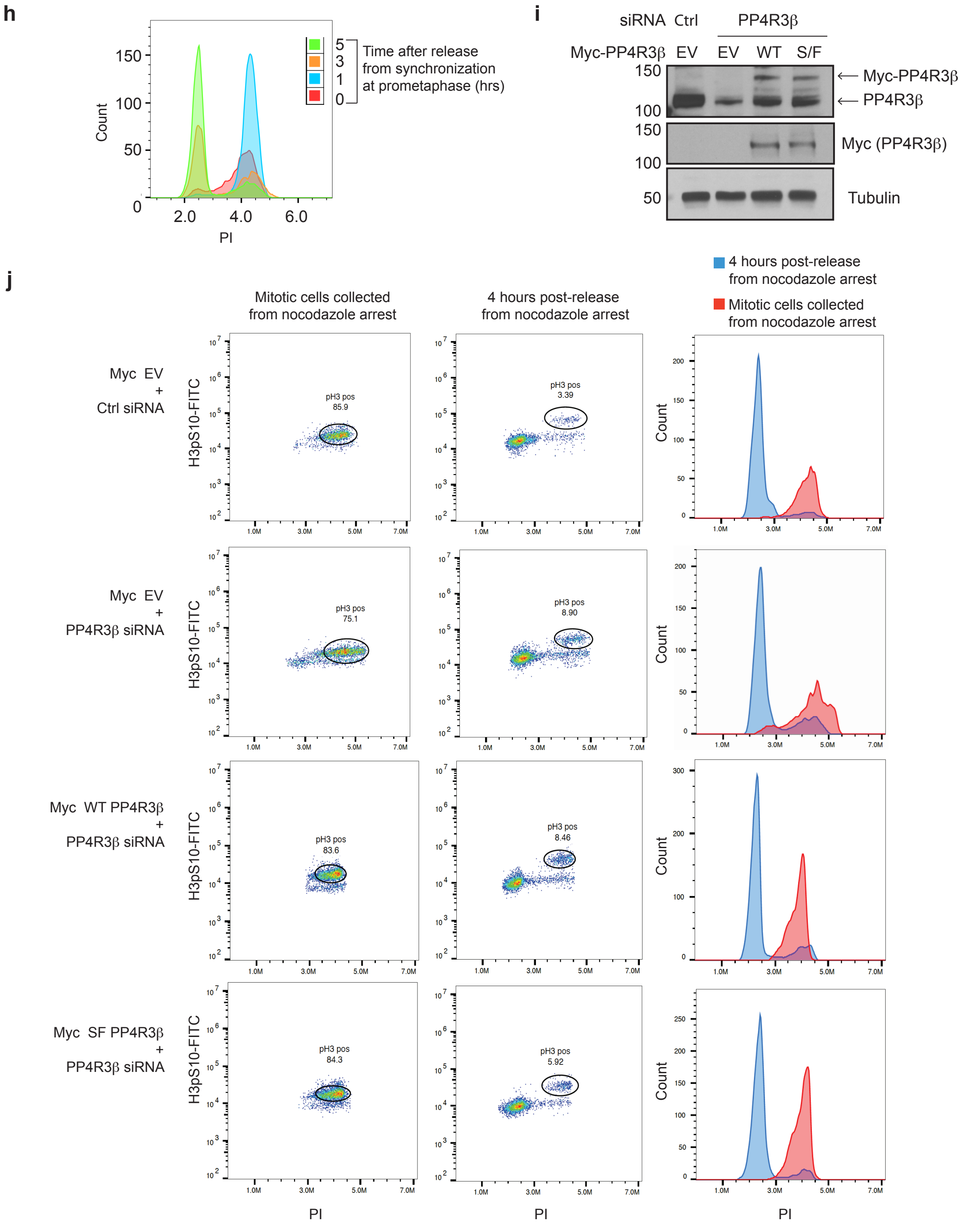

**Supplementary Figure 1 (Related to Figure 1): PP4R3β S840 is a conserved residue that is phosphorylated in mitosis.**

**(a)** Mapping of the 53BP1-interacting domain in PP4R3β. Endogenous 53BP1 was immunoprecipitated from nocodazole-arrested 293T cells transiently transfected with Myc empty vector (EV) or Myc-tagged full length PP4R3α, PP4R3β, and indicated truncation mutants of PP4R3β.

**(b)** Silver-stained gel showing immunoprecipitated FLAG-HA (FH) PP4R3β, from soluble nuclear extracts of HeLa cells arrested at prometaphase. Mock-transfected cells are used as control.

**(c)** Verification of sequence assignment of the phosphorylated residue for PP4R3β S840. The MS/MS spectra of tryptic peptide derived from immunoprecipitated FH-PP4Rβ (upper spectrum) purified from mitotic HeLa cell, and of isotope-encoded synthetic analog (lower spectrum) illustrate the alignment of the major b-type (blue) and y-type (red) fragment ions. Peptide sequences above each spectrum are annotated to show phosphorylated residues (green) and sequence-specific fragments detected in each MS/MS spectrum (with blue and red underlines).

**(d)** Sequence alignment of PP4R3β peptides harboring S840 from vertebrate homologues, performed with MUSCLE (Multiple Sequence Comparison by Log- Expectation http://www.ebi.ac.uk/Tools/msa/muscle/).

**(e)** Sequence alignment of PP4R3α and PP4R3β C-termini show that S840 is a site unique to PP4R3β, performed with CLC Sequence Viewer (https://www.qiagenbioinformatics.com/products/clc-sequence-viewer/).

**(f)** Gating strategy used in flow cytometry analyses. Representative plots of asynchronous HeLa cells: i) Cells were first gated on a forward scatter (FSC-H)/side scatter (SSC-H) plot. ii) Cells were next gated on a propidium iodide (PI)-H/PI-A plot to eliminate doublets. (iii) Finally, the percentage of histone H3 phospho-Ser10 (H3pS10)-positive (pH3 pos) cells was quantified from a H3pS10-FITC/PI-A plot.

**(g)** FACS plots of histone H3 phospho-Ser10 (H3pS10) and propidium iodide (PI) staining of HeLa cells expressing FLAG-53BP1 and indicated Myc-PP4R3β S840 phospho-variants. Cells were either not treated (Asynchronous) or arrested in prometaphase by nocodazole.

**(h)** FACS plot of propidium iodide (PI) staining of HeLa cells, collected at indicated time points after release from RO-3306-induced synchronization at G2/M (related to Figure 1b, c; Figure 2h).

**(i)** Immunoblots comparing the expression levels of endogenous PP4R3β and exogenously-expressed Myc-PP4R3β S840 phospho-variants.

**(j)** FACS plots of histone H3 phospho-Ser10 (H3pS10) and propidium iodide (PI) staining of HeLa cells transfected with indicated siRNAs and complemented with indicated Myc-PP4R3β S840 phospho-variants. Transfected cells were collected from nocodazole-induced prometaphase arrest or at 4 hours after release from nocodazole (related to Figure 1c-e).

## Supplementary figure 2

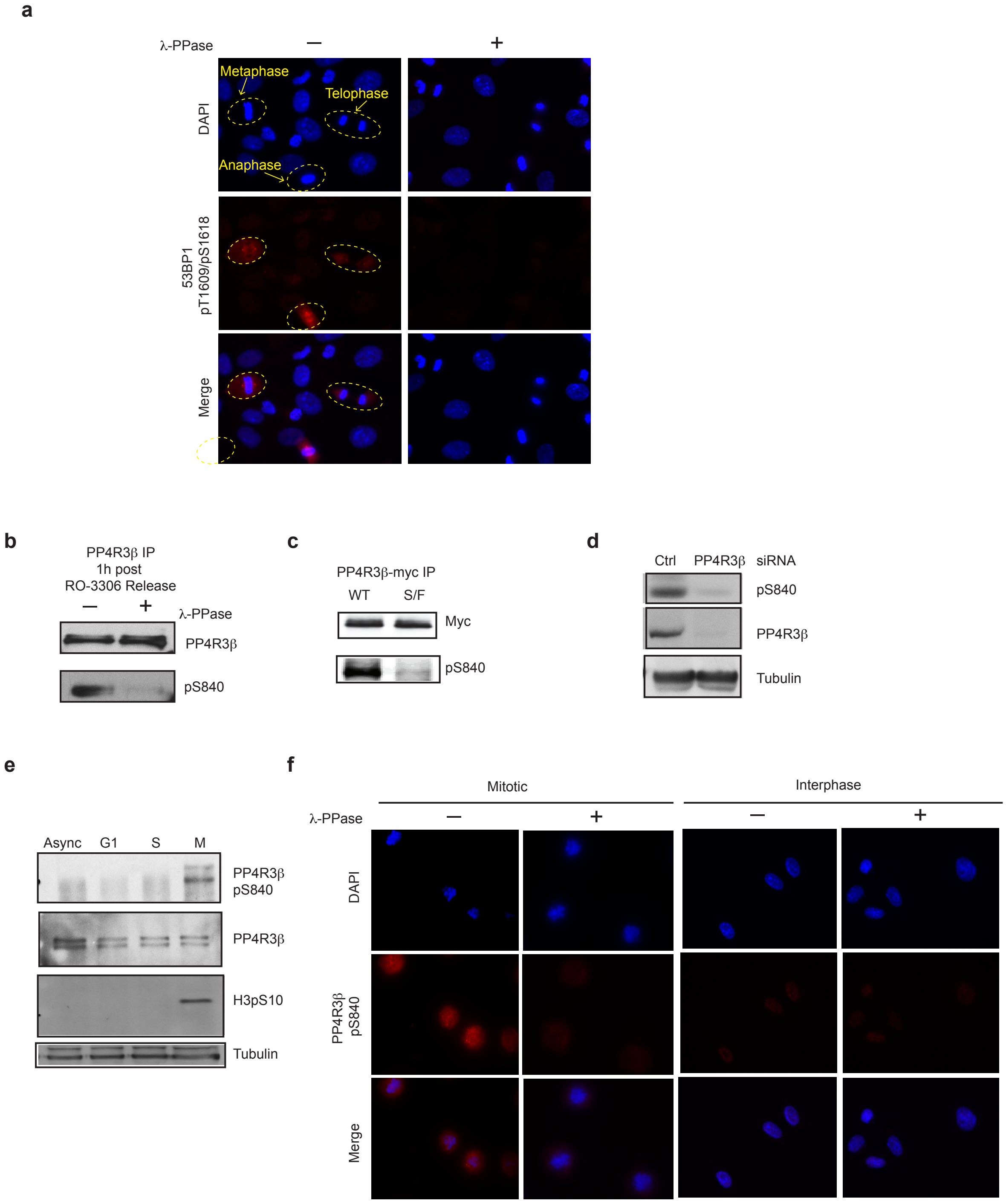

**Supplementary Figure 2 (Related to Figure 2): Phospho-antibodies specifically recognize 53BP1 T1609/S1618 and PP4R3β S840 in mitotic cells.**

- **(a)** Staining of phosphorylated 53BP1 T1609/S1618 in mitotic cells. RPE1 cells were fixed and stained with phospho-specific antibody against T1609/S1618 (pT1609/pS1618) at one hour after release from RO-3306-induced arrest at G2/M. Lack of immunofluorescence signal upon the addition of lambda protein phosphatase (λ-PPase) ensures specificity of the phospho-antibody. Cells at distinct stage of mitosis are outlined in yellow.
- **(b)** Specificity of antibody against PP4R3β phospho-S840 (pS840). Prometaphase U2OS cells were harvested by shake-off and lysed at one hour after release from RO-3306-induced arrest at G2/M. Endogenous PP4R3β was immunoprecipitated from the untreated control or λ-PPase-treated lysate and analyzed by immunoblot.
- **(c)** Phospho-antibody against PP4R3β phospho-S840 (pS840) does not recognize PP4R3β phosphonull variant S840F. U2OS cells were transfected with Myc-PP4R3β WT or S840F. The transfected cells were synchronized to G2/M using RO-3306. One hour after release from RO-3306, prometaphase cells were collected by shake-off and lysed. Myc-PP4R3B WT and S840F were immunoprecipitated by anti-c-Myc agarose and analyzed by immunoblot.
- **(d)** Specificity of antibody against PP4R3β phospho-S840 (pS840). U2OS cells were transfected with either scrambled control (Ctrl) siRNA or siRNA targeting PP4R3β. 24 hours post transfection of siRNAs, the cells were treated with RO3306 for 16 hours. One hour after release from RO-3306, cells were harvested, lysed and analyzed by immunoblot.

**(e)** Phosphorylation of PP4R3β at S840 throughout the cell cycle. HeLa cells were synchronized to mitotic prometaphase using nocodazole (M), 5 hours after release from nocodazole-mediated arrest (G1), and S phase by double thymidine. Endogenous PP4R3β was immunoprecipitated from cells collected at these synchronization times and analyzed for S840 phosphorylation by immunoblot.

**(f)** Phosphorylation of PP4R3β S840 is enriched in mitotic cells compared to interphase cells. HeLa cells, arrested to prometaphase (mitotic) using nocodazole, or not treated (interphase), were fixed and stained with antibody against PP4R3β phospho-S840 (pS840).

## Supplementary figure 3 a-g

**a**

| Site | Kinase motif | Motif Group | Score |
|---|---|---|---|
| S840 | CDK1 kinase | Proline-dependent Ser/Thr kinase | 0.3410 |
| S840 | CDK5 kinase | Proline-dependent Ser/Thr kinase | 0.2468 |

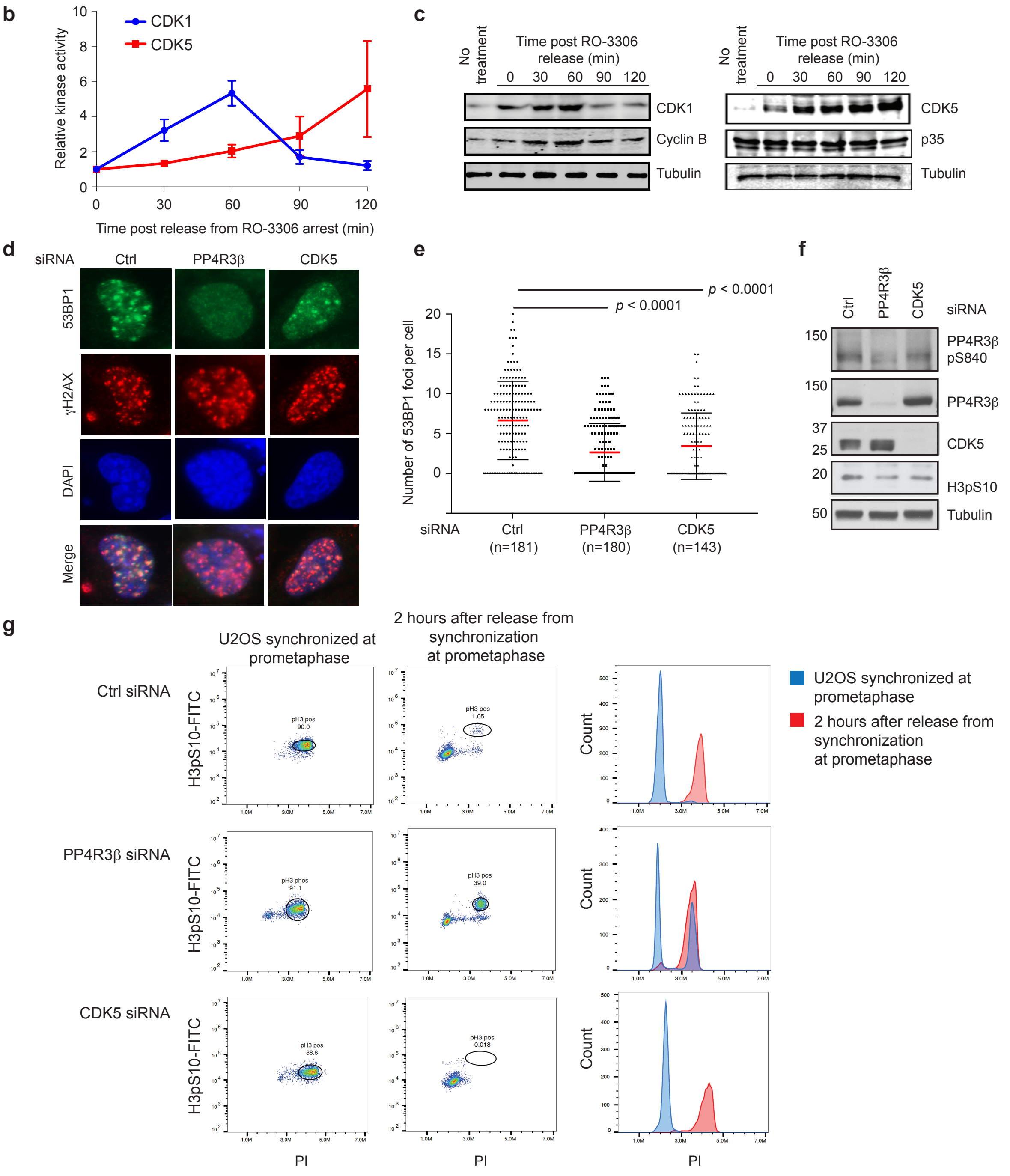

**Supplementary figure 3 h-i**

**h**

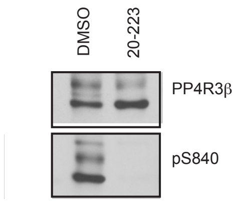


**i**

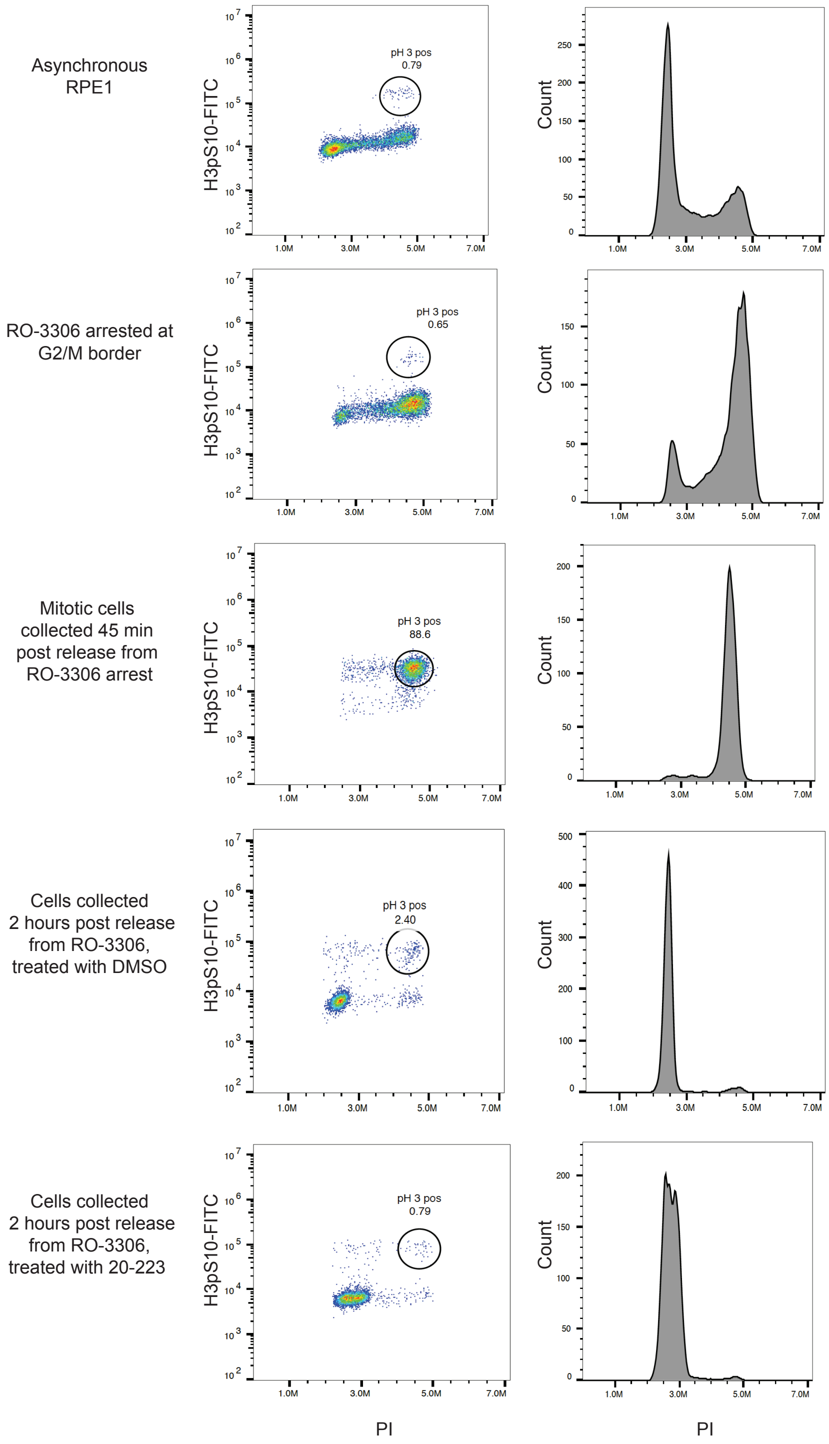

**Supplementary Figure 3 (Related to Figure 2 and Figure 3): CDK5 is active mitosis and required for irradiation-induced 53BP1 foci formation in G1.**

- **(a)** Scansite 3.0 (scansite.mit.edu) prediction of putative kinases that phosphorylate S840 in PP4R3β, based on known kinase recognition motifs. Scores start at 0.000 if the sequences optimally match known kinase motifs and increase as sequences diverge from the optimal match.
- **(b)** Relative kinase activity level of CDK1 across mitosis. HeLa cells harvested at indicated time points after release from RO-3306-mediated arrest at G2/M. Data are expressed as mean ± s.d; n= 3.
- **(c)** Representative immunoblots of indicated proteins present in cell lysates used for kinase activity assay in (b).
- **(d)** Depletion of PP4R3β abrogates irradiation-induced 53BP1 foci formation in G1. U2OS cells were transfected with indicated siRNAs. Transfected cells were synchronized to G2/M using RO-3306. Prometaphase cells were collected by shake-off at one hour after release from RO-3306 arrest and seeded on poly-D-lysine-coated coverslips. Two hours after release from RO-3306-induced arrest, cells were irradiated with 5 Gy. Two hours after irradiation, cells were fixed and stained for indicated antibodies and analyzed by immunofluorescence.
- **(e)** Quantification of 53BP1 foci in (d) from number of cells indicated in parenthesis, pooled from triplicate repeat of experiments. Data are expressed as mean ± s.d. *P*-values, Mann-Whitney U Test.
- **(f)** Immunoblots indicating effective knockdown of PP4R3β and CDK5 in U2OS for Supplementary Fig 3d, e.

**(g)** FACS plots of histone H3 phospho-Ser10 (H3pS10) and propidium iodide (PI) staining of U2OS cells transfected with indicated siRNAs shown in Supplementary Figure 3d, e. Transfected cells were synchronized to G2/M by 16-hour treatment with RO-3306. Prometaphase cells were collected at one hour after release from RO-3306-induced arrest and labeled with H3pS10 and propidium iodide for analysis. Unlabeled prometaphase cells were released for 2 hours longer to proceed into G1 before labeling with H3pS10 and propidium iodide for analysis.

**(h)** Immunoblots indicating abrogation of PP4R3β S840 phosphorylation due to inhibition of CDK5 in RPE1 cells treated with 20-223.

**(i)** FACS plots of histone H3 phospho-Ser10 (H3pS10) and propidium iodide (PI) staining of asynchronous RPE1 cells, RPE1 cells synchronized to G2/M by RO-3306 treatment, prometaphase RPE1 collected by shake-off at 45 minutes after release from RO-3306-induced G2/M arrest, DMSO-treated RPE1 cells at 2 hours after release from RO-3306 arrest, and 20-223-treated RPE1 cells at 2 hours after release from RO-3306 arrest (related to Figure 3 c, d).

## Supplementary figure 4 a-g

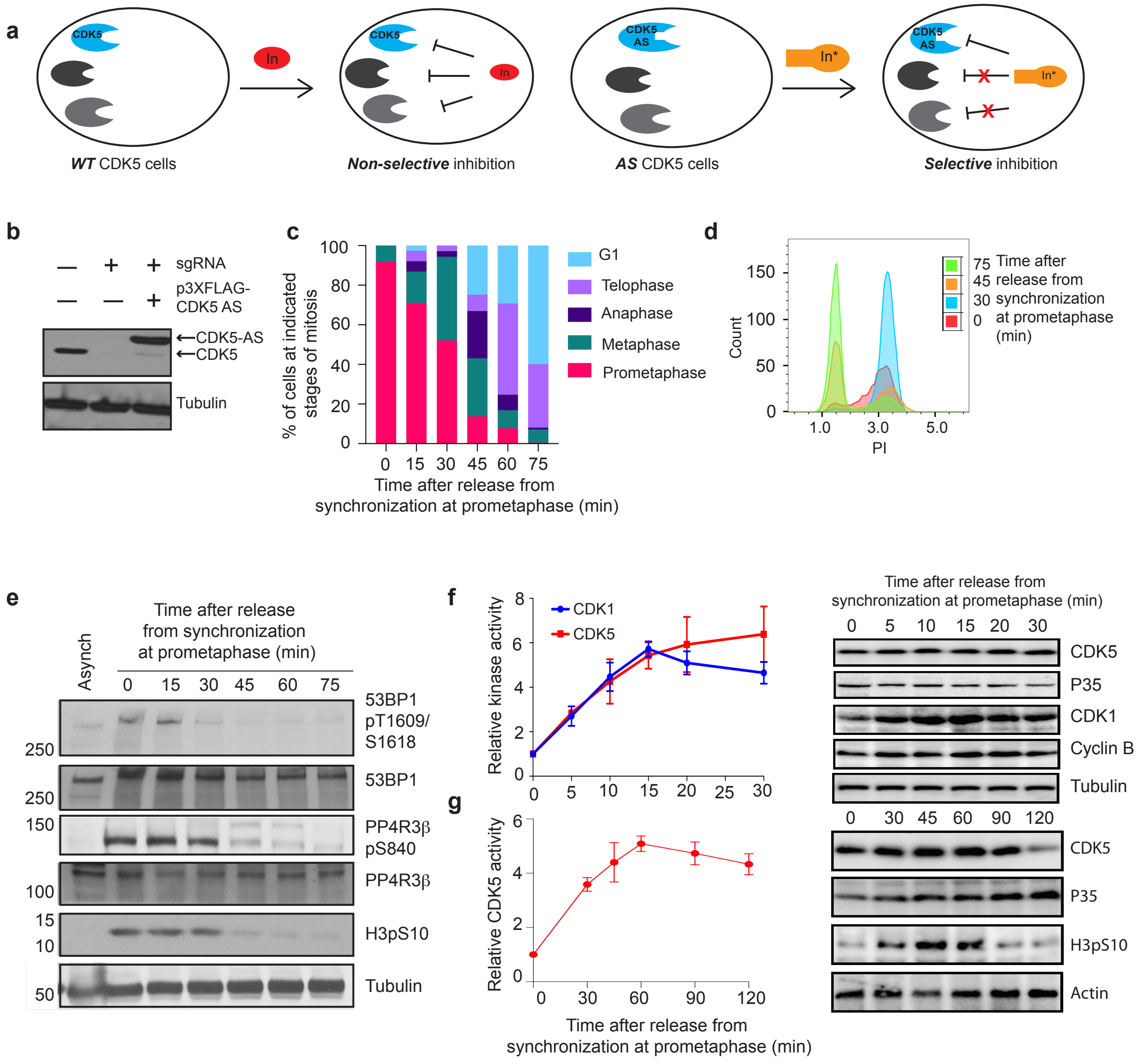

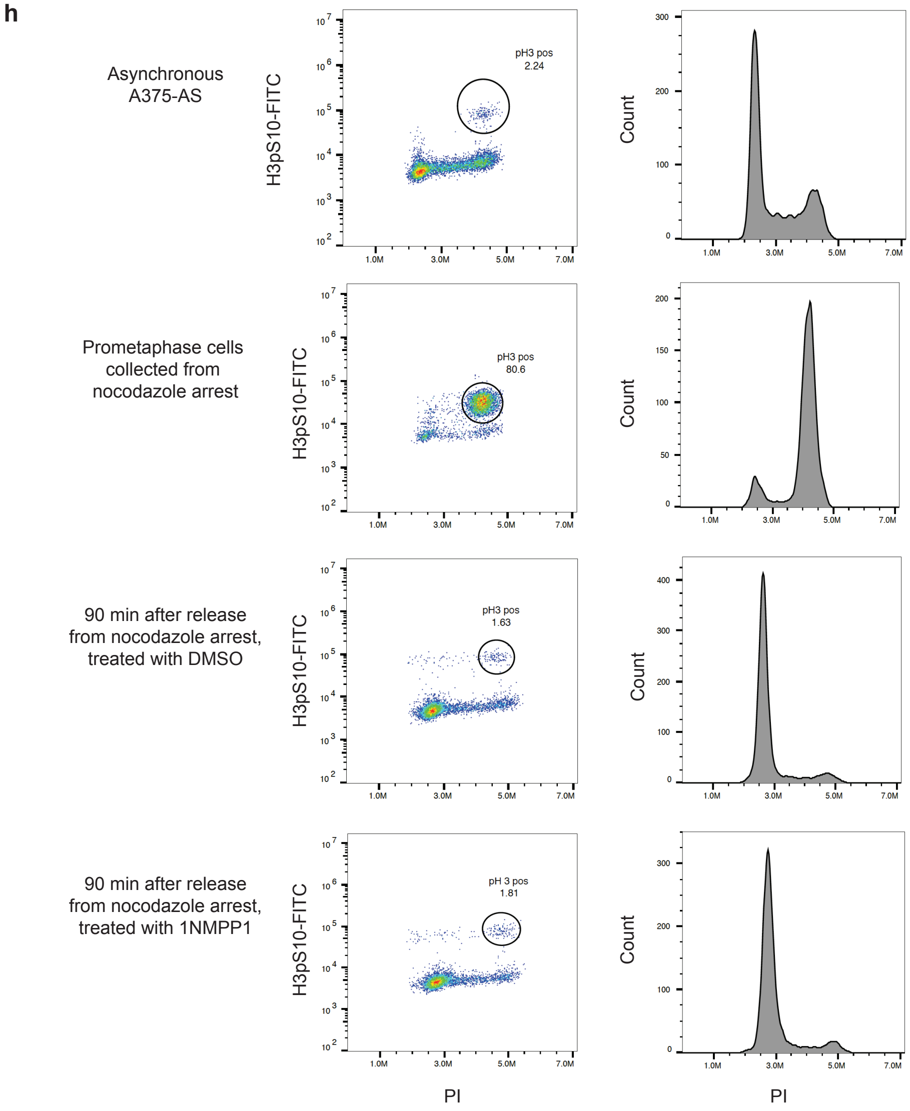
h
Asynchronous
A375-AS
Prometaphase cells
collected from
nocodazole arrest
90 min after release
from nocodazole arrest,
treated with DMSO
90 min after release
from nocodazole arrest,
treated with 1NMPP1
H3pS10-FITC
Count
pH3 pos
2.24
pH3 pos
80.6
pH3 pos
1.63
pH 3 pos
1.81
PI
PI

**Supplemental Figure 4 (Related to Figure 3 and Figure 4). Chemical genetic system for specific inhibition of CDK5.**

**(a)** Schematic of the chemical genetic strategy for specific inhibition of CDK5. Canonical ATP-analog inhibitor (In) targets endogenous CDK5 (WT) at its ATP-binding catalytic site nonspecifically, since multiple kinases share structurally similar catalytic sites (left panel). The analog-sensitive (AS) phenylalanine-to-glycine (F80G) mutation confers a structural change adjacent to the catalytic site of CDK5 that does not impact its catalysis but accommodates the specific binding to a nonhydrolyzable bulky ATP analog 1NMPP1(In*). Hence, CDK5-AS variant can be selectively inhibited by the addition of 1NMPP1 (right panel).

**(b)** Immunoblots indicating CRISPR knockout of endogenous CDK5 in A375 cells and stable expression of FLAG-CDK5 harboring analog-sensitive (AS) mutation F80G.

**(c)** Distribution of A375-AS cells across distinct stages of mitosis at indicated time points after release from synchronization at prometaphase. A375-AS cells were synchronized to prometaphase by treatment with nocodazole. Prometaphase cells were collected by shake-off, seeded onto poly-D-lysine-coated coverslips, and released to progress through mitosis into G,1 and fixed at indicated time points. Cells corresponding to stages of mitosis were quantified based on chromatin morphology, as indicated by DAPI staining.

**(d)** FACS plot of propidium iodide (PI) staining of A375-AS cells, collected at indicated time points after release from nocodazole-induced synchronization at prometaphase (related to Figure 3 g, h).

**(e)** Kinetics of 53BP1 T1609/S1618 phosphorylation and PP4R3β S840 phosphorylation throughout mitosis. A375-AS cells, collected at indicated time points after release from synchronization at prometaphase induced by nocodazole, and analyzed by immunoblot.

**(f)** Kinase activity levels of CDK1 and CDK5 in mitosis and G1. A375-AS cells were synchronized to prometaphase by nocodazole and released to proceed into G1. CDK1 or CDK5 was immunoprecipitated from cells collected at indicated time points after release, and incubated with peptide substrate and [$^{32}$P]-ATP. Radioactivity of labeled substrate peptides were measured in a liquid scintillation counter. Data are expressed as mean ± s.d; n= 3. Representative immunoblots show indicated proteins present in the cell lysate for the kinase assay.

**(g)** Same experiment as described above (Supplementary Fig. 3f), but cells were collected at later time points to assess activity in mid-G1. Data are expressed as mean ± s.d; n= 3. Representative immunoblots show indicated proteins present in the cell lysate for kinase assay.

**(h)** FACS plots of histone H3 phospho-Ser10 (H3pS10) and propidium iodide (PI) staining of: i) asynchronous A375-AS cells, ii) prometaphase cells collected from nocodazole-induced arrest, iii) DMSO-treated cells at 90 minutes after release from nocodazole-induced arrest, and iv) 1NMPP1-treated cells at 90 minutes after release from nocodazole-induced arrest (related to Figure 3 g, h; and Figure 4 c, d).

# Supplementary figure 5

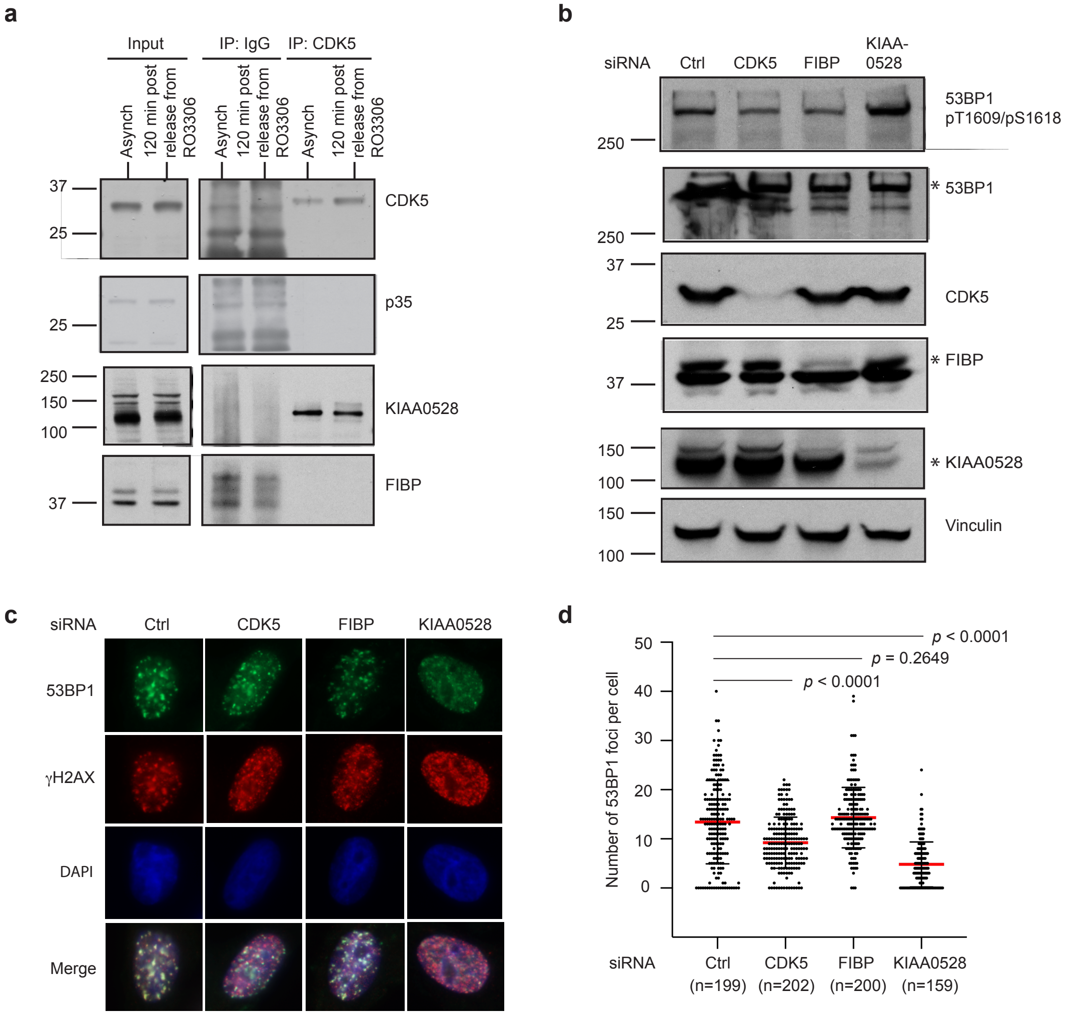

**Supplementary Figure 5. CDK5 binding partner KIAA0528 is required for the recruitment of 53BP1 to DNA damage in non-neuronal cells.**

**(a)** Immunoblots of indicated proteins in the elution of immunoprecipitated endogenous CDK5 from asynchronous RPE1 cells, and from RPE1 cells collected at 120 minutes after release from RO3306-induced G2/M arrest.

**(b)** Immunoblots of indicated proteins in cells treated with indicated siRNAs. Asterisks (*) indicate specific protein bands. HeLa cells transfected with indicated siRNAs were arrested in prometaphase using nocodazole. Mitotic cells were collected by shake-off and released into fresh medium. One hour after release from nocodazole-mediated arrest, cells were collected and lysed for immunoblot.

**(c)** Impact of depletion of CDK5 and binding partners on irradiation-induced 53BP1 foci formation. HeLa cells were transfected with indicated siRNAs and arrested in prometaphase using nocodazole. Prometaphase cells were collected by shake off and seeded onto poly-D-lysine-coated coverslips. Four hours after release from nocodazole-mediated arrest, cells were irradiated with 5 Gy. Two hours after irradiation, cells were fixed and stained with indicated antibodies.

**(d)** Quantifications of 53BP1 foci in (c), pooled from triplicate repeat experiments, expressed as mean ± s.d. Total number of cells is indicated in parenthesis. *P*-values, Mann-Whitney U Test.

**Supplementary Table 1. List of primers used in this study.**

| # | Primer | Sequence | Purpose |
|---|---|---|---|
| 1 | R3B FL Fwd-NotI-Myc | 5' TTT TGC GGC CGC TAT GTC GGA TAC GCG GCG | Clone full length PP4R3β into pCDNA3.1-HisA-6xMyc |
| 2 | R3B FL Rev-XbaI-Myc | 5' TTT TTC TAG A TT A TG AGC CAA GAC GAG GTC TTT TCC | Clone full length PP4R3β into pCDNA3.1-HisA-6xMyc |
| 3 | R3B C5 Fwd-XhoI-Myc | 5' TTT TCT CGA GTC GAA GAA GAG GAA GGA AAA GCA GTT G | Clone C-terminal PP4R3β fragment aa721-849 into pCDNA3.1-HisA-6xMyc |
| 4 | R3B 400 Rev-XbaI-Myc | 5' TTT TTC TAG ATT ACT CTC GGA CCA TAG ATG GAC TAA ATT C | Clone N-terminal PP4R3β fragment aa1-400 into pCDNA3.1-HisA-6xMyc |
| 5 | R3B 700 Rev-Xba-Myc | TTT TTC TAG ATT AGT ACC ATC TAT ATT GCG TAG TAA C | Clone N-terminal PP4R3β fragment aa1-700 into pCDNA3.1-HisA-6xMyc |
| 6 | SMEK2 S840A Fwd | 5' GAT GAA GAA GAA GAA TCG GCC CCC AGG AAA AGA CC | Site-direct mutagenesis to mutate PP4R3β/SMEK2 S840 to S840A |
| 7 | SMEK2 S840A Rev | 5' GGT CTT TTC CTG GGG GCC GAT TCT TCT TCT TCA TC | Site-direct mutagenesis to mutate PP4R3β/SMEK2 S840 to S840A |
| 8 | SMEK2 S840F Fwd | 5' GAT GAA GAA GAA GAA TCG TTC CCC AGG AAA AGA CC | Site-direct mutagenesis to mutate PP4R3β/SMEK2 S840 to S840F |
| 9 | SMEK2 S840F Rev | 5' GGT CTT TTC CTG GGG AAC GAT TCT TCT TCT TCA TC | Site-direct mutagenesis to mutate PP4R3β/SMEK2 S840 to S840F |
| 10 | SMEK2 S840D Fwd | 5' GAT GAA GAA GAA GAA TCG GAC CCC AGG AAA AGA CC | Site-direct mutagenesis to mutate PP4R3β/SMEK2 S840 to S840D |
| 11 | SMEK2 S840D Rev | 5' GGT CTT TTC CTG GGG TCC GAT TCT TCT TCT TCA TC | Site-direct mutagenesis to mutate PP4R3β/SMEK2 S840 to S840D |
| 12 | SMEK2 XhoI pET15 Fwd | 5' AAT GCT CGA GTC GGA TAC GCG GCG GCG A | Clone PP4R3β/SMEK2 into pET15B |
| 13 | SMEK2 XhoI pET15 Rev | 5' GAT CCT CGA GTT ATG AGC CAA GAC GAG GTC | Clone PP4R3β/SMEK2 into pET15B |
| 14 | R3B ORFsi res SDM Fwd | 5' CAG AAA CTG AAC AGT GTA CCC TCC ATC CTC CGG AGT AAC AGA TTT CGC AGA G | Site-directed mutagenesis to generate siRNA-resistant constructs against PP4R3β siRNA #1 |

| | | | |
|---|---|---|---|
| 15 | R3B ORFsi res SDM Rev | 5’ CTC TGC GAA ATC TGT TAC TCC GGA GGA TGG AGG GTA CAC TGT TCA GTT TCT G | Site-directed mutagenesis to generate siRNA-resistant constructs against PP4R3β siRNA #1 |
| 16 | pOZ FH R3B FL Fwd XhoI | 5’ TTT TCT CGA GAT GTC GGA TAC GCG GC | Clone PP4R3β full length into pOZ-FLAG-HA |
| 17 | POZ FH R3B FL Rev NotI | 5’ TTT TGC GGC CGC TTA TGA GCC AAG ACG AGG TCT TT | Clone PP4R3β full length into pOZ-FLAG-HA |

**Supplementary Table 2. List of antibodies used in this study**

| Target | Manufacturer | Catalogue number | Host | Dilution or amount used | Application |
|---|---|---|---|---|---|
| p35/25 | Cell Signaling Technology | 2680 | Rabbit | 1:500 | Immunoblotting |
| 53BP1 | Santa Cruz | sc-22760 | Rabbit | 5ug per mg lysate | Immunoprecipitation |
| 53BP1 | Santa Cruz | sc-22760 | Rabbit | 1:500 | Immunoblotting, immunofluorescence |
| 53BP1 | Novus Bio | NB100-304 | Rabbit | 1:500 | Immunofluorescence |
| 53BP1 Phospho-T1609/S1618 | Antagene | Custom made | Rabbit | 1:100 | Immunoblotting, Immunofluorescence |
| Alpha-tubulin | Sigma | T6074 | Mouse | 1:4000 | Immunoblotting |
| c-Myc (9E10) | Biolegend | 658502 | Mouse | 15ul slurry per mg lysate | Immunoprecipitation |
| c-Myc | Santa Cruz | sc-40 | Mouse | 1:1000 | Immunoblotting |
| c-Myc | Santa Cruz | sc-789 | Rabbit | 1:1000 | Immunoblotting |
| CDK1 | Abcam | ab133327 | Rabbit | 1:1000 | Immunoblotting |
| CDK5 | Santa Cruz | sc-173 | Rabbit | 1:500 | Immunoblotting |
| CDK5 | Santa Cruz | sc-6247 | Mouse | 1:500 | Immunoblotting |
| CDK5 | Santa Cruz | sc-249 AC | Mouse | 15 µl slurry per mg lysate | Immunoprecipitation |
| Cyclin A | Santa Cruz | sc-271682 | Mouse | 1:100 | Immunofluorescence |
| Cyclin B1 | Santa Cruz | sc-245 | Mouse | 1:100 | Immunoblotting |
| FIBP | EpiGentek | A50225 | Rabbit | 1:500 | Immunoblotting |
| FLAG | Sigma-Aldrich | F1804 | Mouse | 1:1000 | Immunoblotting |
| FLAG | Sigma-Aldrich | F7425 | Rabbit | 1:1000 | Immunoblotting |
| FLAG (M2) | Sigma-Aldrich | A2220 | Mouse | 15ul slurry per mg lysate | Immunoprecipitation |
| KIAA0528 | Bethyl | A301-469A | Rabbit | 1:1000 | Immunoblotting |
| Phospho-Histone H2AX (Ser139) | EMD Millipore | 05-636 | Mouse | 1:500 | Immunofluorescence |

| | | | | | |
|---|---|---|---|---|---|
| Phospho-Histone H3 (Ser 10) | Cell Signaling | 9701 | Rabbit | 1:1000 | Immunoblotting |
| Phospho-Histone H3 (Ser 10) AlexaFluor 488 conjugated | EMD Millipore | 06-570-AF488 | Rabbit | 1:200 | Flow cytometry |
| PP4C | Bethyl | A300-835A | Rabbit | 1:1000 | Immunoblotting |
| PP4R3β Phospho-S840 | GL Biochem Ltd | Custom made | Rabbit | 1:80 | Immunoblotting, Immunofluorescence |
| PP4R3β | Bethyl | A300-842A | Rabbit | 1:1000 | Immunoblotting |
| Protein A/G PLUS agarose | Santa Cruz | Sc-2003 | N/A | 20 µl slurry per mg lysate | Immunoprecipitation |
| Vinculin | Santa Cruz | sc-25336 | Mouse | 1:2000 | Immunoblotting |
| Alexa Fluor™ 488 anti-mouse IgG | Invitrogen | A11001 | Goat | 1:1000 | Immunofluorescence |
| Alexa Fluor™ 488 anti-rabbit IgG | Invitrogen | A11034 | Goat | 1:1000 | Immunofluorescence |
| Alexa Fluor™ 594 anti-mouse IgG | Invitrogen | A21203 | Donkey | 1:1000 | Immunofluorescence |
| Alexa Fluor™ 594 anti-rabbit IgG | Invitrogen | A21207 | Donkey | 1:1000 | Immunofluorescence |
