# Supplementary material for: A mitotic CDK5-PP4 phospho-signaling cascade primes 53BP1 for DNA repair in G1
Source: Nat Commun. 2019 Sep 18;10:4252. doi: 10.1038/s41467-019-12084-x (PMC6751209; doi:10.1038/s41467-019-12084-x)
Supplement: Supplementary file 3 — Reporting Summary [file 41467_2019_12084_MOESM3_ESM.pdf]

nature research

Corresponding author(s): Dipanjan Chowdhury

Last updated by author(s): Jun 26, 2019

Reporting Summary

Nature Research wishes to improve the reproducibility of the work that we publish. This form provides structure for consistency and transparency in reporting. For further information on Nature Research policies, see Authors & Referees and the Editorial Policy Checklist.

Statistics

For all statistical analyses, confirm that the following items are present in the figure legend, table legend, main text, or Methods section.

n/a	Confirmed	
☐	☒	The exact sample size (*n*) for each experimental group/condition, given as a discrete number and unit of measurement
☐	☒	A statement on whether measurements were taken from distinct samples or whether the same sample was measured repeatedly
☒	☐	The statistical test(s) used AND whether they are one- or two-sided<br>*Only common tests should be described solely by name; describe more complex techniques in the Methods section.*
☒	☐	A description of all covariates tested
☒	☐	A description of any assumptions or corrections, such as tests of normality and adjustment for multiple comparisons
☐	☒	A full description of the statistical parameters including central tendency (e.g. means) or other basic estimates (e.g. regression coefficient) AND variation (e.g. standard deviation) or associated estimates of uncertainty (e.g. confidence intervals)
☐	☒	For null hypothesis testing, the test statistic (e.g. *F*, *t*, *r*) with confidence intervals, effect sizes, degrees of freedom and *P* value noted<br>*Give P values as exact values whenever suitable.*
☒	☐	For Bayesian analysis, information on the choice of priors and Markov chain Monte Carlo settings
☒	☐	For hierarchical and complex designs, identification of the appropriate level for tests and full reporting of outcomes
☒	☐	Estimates of effect sizes (e.g. Cohen's *d*, Pearson's *r*), indicating how they were calculated

Our web collection on statistics for biologists contains articles on many of the points above.

Software and code

Policy information about availability of computer code

Data collection	Zeiss Zen 2.3 Pro was used for immunofluorescence imaging acquisition
Data analysis	GraphPad Prism 7, ImageJ 2.2.0-rc-54/1.51f, Mascot version 2.2, and FlowJo 10.6.0

For manuscripts utilizing custom algorithms or software that are central to the research but not yet described in published literature, software must be made available to editors/reviewers. We strongly encourage code deposition in a community repository (e.g. GitHub). See the Nature Research guidelines for submitting code & software for further information.

Data

Policy information about availability of data

All manuscripts must include a data availability statement. This statement should provide the following information, where applicable:

- Accession codes, unique identifiers, or web links for publicly available datasets
- A list of figures that have associated raw data
- A description of any restrictions on data availability

Data that support the findings of this study are available from the corresponding author upon reasonable request.

Field-specific reporting

Please select the one below that is the best fit for your research. If you are not sure, read the appropriate sections before making your selection.

☒ Life sciences ☐ Behavioural & social sciences ☐ Ecological, evolutionary & environmental sciences

For a reference copy of the document with all sections, see nature.com/documents/nr-reporting-summary-flat.pdf

Life sciences study design

All studies must disclose on these points even when the disclosure is negative.

Sample size	No statistical method was used to predetermine sample size. All experiments were included with multiple biological replicates based on previous experiences.
Data exclusions	No sample were excluded from analysis
Replication	All resulsts were tested and confirmed with at least two independent experiments
Randomization	No method of randomization was applied. Samples were organized into groups based on whether they were treated or untreated (IR, chemical treatment, knockdown by siRNA)
Blinding	No blinding assessment was performed.

Reporting for specific materials, systems and methods

We require information from authors about some types of materials, experimental systems and methods used in many studies. Here, indicate whether each material, system or method listed is relevant to your study. If you are not sure if a list item applies to your research, read the appropriate section before selecting a response.

Materials & experimental systems

n/a	Involved in the study	
☐	☒	Antibodies
☐	☒	Eukaryotic cell lines
☒	☐	Palaeontology
☒	☐	Animals and other organisms
☒	☐	Human research participants
☒	☐	Clinical data

Methods

n/a	Involved in the study	
☒	☐	ChIP-seq
☐	☒	Flow cytometry
☒	☐	MRI-based neuroimaging

Antibodies

Antibodies used

Target/Manufacturer/Catalogue number/Host/Dilution or Amount used/Application- Immunofluorescence (IF), Immunoprecipitation (IP), Western blot (WB), flow cytometry (FC)

p35/25 Cell Signaling Technology #2680 Rabbit 1 to 500 WB
53BP1 Santa Cruz sc-22760 Rabbit 5ug per mg lysate IP
53BP1 Santa Cruz sc-22760 Rabbit 1 to 500 WB, IF
53BP1 NovusBio NB100-304 Rabbit 1 to 500 IF
Alpha-tubulin Sigma T6074 Mouse 1 to 4000 WB
c-Myc (9E10) affinity gel Biolegend 658502 Mouse 15ul slurry per mg lysate IP
c-Myc Santa Cruz sc-40 Mouse 1 to 1000 WB
c-Myc Santa Cruz sc-789 Rabbit 1 to 1000 WB
CDK1 Abcam ab133327 Rabbit 1 to 1000 WB
CDK5 Santa Cruz sc-173 Rabbit 1 to 500 WB
CDK5 Santa Cruz sc-6247 Mouse 1 to 500 WB
CDK5 Santa Cruz sc-249 AC (agarose conjugated) Mouse 15 μl slurry per mg lysate IP
Cyclin A Santa Cruz sc-271682 Mouse 1 to 100 IF
Cyclin B1 Santa Cruz sc-245 Mouse 1 to 100 WB
FIBP EpiGentek A50225 Rabbit 1 to 500 WB
FLAG Sigma-Aldrich F1804 Mouse 1 to 1000 WB
FLAG Sigma-Aldrich F7425 Rabbit 1 to 1000 WB
FLAG (M2) affinity gel Sigma-Aldrich A2220 Mouse 15ul slurry per mg lysate IP
KIAA0528 Bethyl A301-469A Rabbit 1 to 1000 WB
Phospho-Histone H2AX (Ser139) EMD Millipore 05-636 Mouse 1 to 500 IF
Phospho-Histone H3 (Ser 10) Cell Signaling #9701 Rabbit 1 to 1000 WB
Phospho-Histone H3 (Ser 10) AlexaFluor 488 conjugated EMD Millipore 06-570-AF488 Rabbit 1 to 200 FC
Phospho-53BP1 T1609/S1618 Antagene Custom made Rabbit 1 to 100 WB, IF
Phospho-PP4R3β S840 GL Biochem Ltd Custom made Rabbit 1 to 80 WB, IF
PP4C Bethyl A300-835A PP4C 1 to 1000 WB
PP4R3β Bethyl A300-842A Rabbit 1 to 1000 WB
Vinculin Santa Cruz sc-25336 Mouse 1 to 2000 WB

Validation

Antibodies are validated with western blot or IF with tagged ectopic expression as positive control and siRNA knockdown as negative control. For gamma H2AX, cells with and without irradiation was used as positive and negative control. For antibodies

against phospho-53BP1 at T1609/S1618 and phospho-PP4R3B at S840, cells treated with or without lambda phosphatase were used as positive and negative controls, respectively.

Eukaryotic cell lines

Policy information about cell lines

Cell line source(s)	293T: ATCC<br>HeLa: ATCC<br>RPE1-TERT: ATCC<br>UWB1.289 :ATCC<br>A375: Dr. Priotr Sicinski<br>A375-CDK5 analog sensitive (AS): Dr. Priotr Sicinski
Authentication	The cell lines have been authenticated based on morphological criteria.
Mycoplasma contamination	All cell lines were tested negative for mycoplasma.
Commonly misidentified lines<br>(See ICLAC register)	No commonly misidentified cell lines were used

Flow Cytometry

Plots

Confirm that:

☒ The axis labels state the marker and fluorochrome used (e.g. CD4-FITC).

☒ The axis scales are clearly visible. Include numbers along axes only for bottom left plot of group (a 'group' is an analysis of identical markers).

☒ All plots are contour plots with outliers or pseudocolor plots.

☒ A numerical value for number of cells or percentage (with statistics) is provided.

Methodology

Sample preparation	HeLa, RPE1, U2OS, and A375-AS cells were fixed in cold 70% ethanol, processed with reagents from the FlowCellect Histone H2A.X Phosphorylation Assay Kit (EMD Millipore) per manufacturer's instructions, hybridized with Anti-phospho Histone H3 (Ser10) antibody-Alexa Fluor 488 conjugate (EMD Millipore). The genomic DNA was stained with propidium iodide (Sigma, Cat. No. 81845) in combination with RNAse (Roche, Cat. No. 11119915001).
Instrument	Beckman Coulter Cytoflex S
Software	Data were collected with the CytExpert and analyzed with the FlowJo software.
Cell population abundance	10,000 cells per sample were measured and analyzed, out of which 75-80% were live cells that were selected for single cells. Single cells selected for H3pS10 staining analysis were above 90%.
Gating strategy	For all experiments, cells were preliminary gated according to FSC/SSC scatters and PI-peak/ PI-area (only cells on the diagonal were considered to remove cell doublets). The gates used for cell sorting and analyses are shown in Supplementary Figure 1f.

☒ Tick this box to confirm that a figure exemplifying the gating strategy is provided in the Supplementary Information.
